# High-Sensitivity Troponin T and Copeptin in Non-ST Acute Coronary Syndromes: Implications for Prognosis and Role of hsTnT and Copeptin in Non-STEACS

**DOI:** 10.1100/2012/578616

**Published:** 2012-01-04

**Authors:** Diana Hernández-Romero, José María García-Salas, Ángel López-Cuenca, Patricio Pérez-Berbel, Carmen Puche, Teresa Casas, Esteban Orenes-Piñero, Sergio Manzano-Fernández, Mariano Valdés, Francisco Marín

**Affiliations:** ^1^Servicio de Cardiología, Hospital Universitario Virgen de la Arrixaca, 30120 Murcia, Spain; ^2^Servicio de Análisis Clínicos, Hospital Universitario Virgen de la Arrixaca, 30120 Murcia, Spain; ^3^Servicio de Cardiología, Hospital General Universitario de Alicante, 03010 Alicante, Spain

## Abstract

High-sensitivity TnT (hsTnT) has been proposed to improve the diagnosis and stratification in acute coronary syndromes. Copeptin has been proposed for a rapid and accurate rule out of acute myocardial infarction, but some doubts exist about its use out of the first hours from admission. Abnormalities of serum hsTnT and copeptin levels in non-STEACS and negative TnT, could have prognostic implications. *Methods*. We included 122 non-STEACS patients without raised TnT, 33 disease controls and 43 healthy controls. We measured hsTnT and copeptin levels. Clinical follow-up at 12 months was performed for adverse endpoints. *Results*. Non-STEACS patients had raised hsTnT compared with both control groups (*P* = 0.036 and *P* < 0.001). Copeptin levels were higher in non-STEACS patients than healthy controls (*P* = 0.021), without differences with disease controls. Raised levels of hs-TnT presented prognostic implications [HR 3.29 (95%CI: 1.33–7.49), *P* = 0.010]. hs-TnT could be used for invasive approach decision, as it shows prognostic relevance in conservative approach-patients whereas remains unrelevant for catheterized-patients. Copeptin levels were not associated with adverse events. *Conclusion*. hsTnT levels increased in non-STEACS, were predictive of adverse events and could be important for recommending an invasive management. We cannot confirm a predictive role of copeptin out of the first hours from admission.

## 1. Introduction

Acute coronary syndromes (ACSs) present a complex and heterogeneous pathophysiology [1–3] with high morbidity and mortality mainly due to new cardiac ischaemic events [4]. Current stratification of the risk in patients presenting with ACS without ST-segment elevation (non-STEACS) is based on the identification of those patients with higher risk of suffering adverse events (death, recurrent MI or urgent revascularization), estimated in a 15–30% of non-STEACS patients. However, up to 10% of patients who classified as low risk show a cardiovascular event at 3 months followup [5]. Regarding this, the current stratification needs to be improved. It has been proposed to identify new biomarkers or even to use a multimarker approach [6].

Cardiac troponins are components of the contractile apparatus of cardiomyocytes and are released during myocardial necrosis in patients with ACS [7]. Serum troponin (Tn) elevation is a specific and well-established necrosis biomarker in ACS, being the only biomarker currently used for risk stratification and guided invasive management decision in non-STEACS [8, 9]. In patients showing negative Tn elevation, stratification is more complicated and the elective treatment is usually under the criterium of the cardiologist, although there are established recommendations [10, 11]. Conventional determination methods fail to detect slightly increases of Tn, due in part to the delayed and progressive release of the biomarker after the event. It has been shown that even very small elevation in the troponin concentration is associated with increased risk of adverse outcomes in patients with ACS [12]. A recent publication has shown that cardiac troponin T (TnT) concentrations measured with a highly sensitive assay were significantly associated with the incidence of cardiovascular death and heart failure in stable coronary artery disease after adjustment for other independent prognostic indicators [13]. In the same way, Reichlin et al. found that the diagnostic performance of sensitivity cardiac Tn assays is excellent within the context of the myocardial infarction, and these assays can substantially improve the early diagnosis of acute myocardial infarction, particularly in patients with a recent onset of chest pain [14]. However, the criteria defining a high sensitive TnT (hsTnT) assay are still under debate, whereas a cutoff point of TnT for risk stratification in patients with ACS remains difficult to establish due to the heterogeneity of the used detection techniques among different laboratories [15].

As the released of the necrosis markers after cell damage might explain the weakness in diagnostic performance of conventional Tn assays early after chest pain onset, markers with different pathophysiologic background independent of cell necrosis might help in the diagnosis of cardiovascular diseases [16]. Arginine vasopressin (AVP), also known as the antidiuretic hormone, is a peptide secreted neurohypophyseal hormone and controls osmotic homeostasis [17]. However, vasopressin is difficult to measure because it is unstable and rapidly cleared [18]. Copeptin, a 39-amino acid glycopeptides that comprises the C-terminal part of the AVP precursor, was found to be a stable and sensitive surrogate marker of AVP release. Copeptin is secreted in equimolar amounts, and it can be quickly and reliably measured in unprocessed plasma or serum. The predictive value of this marker has been shown in coronary artery disease [19], and it has been proposed to be of great value in the rapid rule out of acute myocardial infarction used in combination with TnT increased levels [20].

We hypothesized that the use of a marker of cardiac necrosis determined by high sensitivity methods, such as hsTnT, or a pathophysiologically different biomarker reflecting acute endogenous stress, such as copeptin, together with the association to the classical clinical and electrocardiographic parameters, might help to the better stratification and managements as well as in the prognosis of patients with non-STEACS.

## 2. Methods

### 2.1. Patients Admission and Selection

We prospectively recruited consecutive patients admitted with a final diagnosis of non-STEACS in two tertiary hospitals. Blood samples were collected for all patients within 48 hours of hospital admission, and baseline clinical characteristics were prospectively recorded. Serum TnT was measured at admission using a commercially available third-generation immunoassay (Elecsys troponin T STAT; Roche Diagnostics, Mannheim, Germany). According to the manufacturers, the lower limit of detection and the concentration with ≤10% precision were 0.01 and 0.035 ng/mL for TnT. We adopted the recommendations of the Joint Committee of the American College of Cardiology and the European Society of Cardiology [21] to establish the upper limit of normal cutoff value of 0.035 ng/mL for TnT. All patients included were stratified as intermediate or high risk by 2002 ACC/AHA Guidelines for Diagnostic and Treatment Strategies in the Emergency Department for Patients with ACS [22]. Patients with elevated conventional TnT (TnT >0.035 mg/mL), concomitant neoplastic, infectious, connective tissue, or inflammatory diseases were excluded (*n* = 74).

During the entire hospitalization period, all patients received standard management as recommended for ACS [10, 23] and clinical management decisions about each patient were decided by the cardiologist responsible, who was unaware of the patient's hsTnT and copeptin concentrations.

Patients with non-STEACS were compared with a group of subjects with stable coronary artery disease (CAD), defined as patients with previous (>6 months) ST elevation myocardial infarct, non-ST elevation myocardial infarct or stable angina, without anginal symptoms for at least 2 months or negative treadmill test at the same time, recruited from consecutive subjects in our outpatient clinics. In addition, we recruited “healthy controls” consisted of hospital staff and relatives, who were defined as “healthy” by careful clinical history and physical examination, ECG, and routine blood tests. The Research Ethics Commitee of our two centres approved the study, and all the subjects gave informed consent to participation.

### 2.2. Blood Samples and Laboratory Assays

Venepuncture was performed within 48 hours of hospital admission, usually the next morning with the patient fasting for >12 hours. In patients with stable CAD and healthy controls, blood samples were taken after overnight fasting and abstinence from tobacco, alcoholic, or caffeine-containing beverages the evening before. Serum fractions were obtained by centrifugation for 15 minutes at 3500 g. Aliquots were stored at −40°C to allow batch analysis in a blinded fashion. Serum levels of hsTnT were assayed by a Cobas 6000 analyser (Roche Diagnostics, Mannheim, Germany). The interassay variation for hsTnT determining was 2.4%, with a lower detection limit of 0.003 ng/mL. We used the established hsTnT cutoff point in ACS in the literature, 0.013 ng/mL [24]. We also determined copeptin in serum by sandwich immunoluminometric assay (CT-proAVP LIA B.R.A.H.M.S. AG, Hennigsdor, Germany). The detection limit was 0.4 pmol/L and the interassay variation coefficient <20%.

### 2.3. Followup and Adverse Clinical Endpoints

All patients were followed for 12 months by outpatient clinic attendance, telephone contact, and review of the medical notes. We defined “adverse clinical endpoints” as cardiovascular death (death in context of ischaemic or other heart disease, of death with unexplained cause, presumed cardiac), nonfatal MI, unstable angina and stroke, and/or admission for acute heart failure.

### 2.4. Statistical Analysis

Continuous variables were tested for normal distribution by the Kolmogorov-Smirnov test. The normal distributed continuous variables are shown as mean ± standard deviation, and those nonparametrically distributed are shown as median (interquartile range). Categorical variables are presented as frequencies (percentages). Comparisons of the groups for continuous variables were performed with the unpaired *t*-test for independent samples or the Mann-Whitney *U* test (as appropriate). The comparison of discrete variables was done by *χ* ^2^ test or Fisher test (as appropriate). Correlation between two continuous variables was performed using the Spearman test. An ANOVA test (if relevant a Kruskall Wallis test) was performed to assess the differences between biological markers and analysed subject groups. In addition, multiple binary logistic regression analyses were used to determine the clinical and biological factors associated with the presence of elevated biomarkers (levels above their cutoff point). The independent effect of variables on prognosis was calculated using a Cox proportional hazards regression model, incorporating in the multivariate model only those variables that showed *P* value < 0.15 in the univariate analysis. The cumulative incidence of adverse clinical events was estimated according to the Kaplan-Meier method, and the log-rank statistic was used for comparisons. A *P* < 0.05 was accepted as statistically significant. Statistical analysis was performed using SPSS 15.0 for Windows (SPSS, Inc., Chicago, IL, USA).

## 3. Results

A total of 198 subjects were included for the analyses: 122 patients with non-STEACS and conventional TnT levels ≤ 0.035 mg/mL, 33 patients with stable CAD, and 43 healthy controls. The distribution of clinical characteristics and laboratory parameters are listed in Table 1.

### 3.1. HsTnT

**Figure 1:**
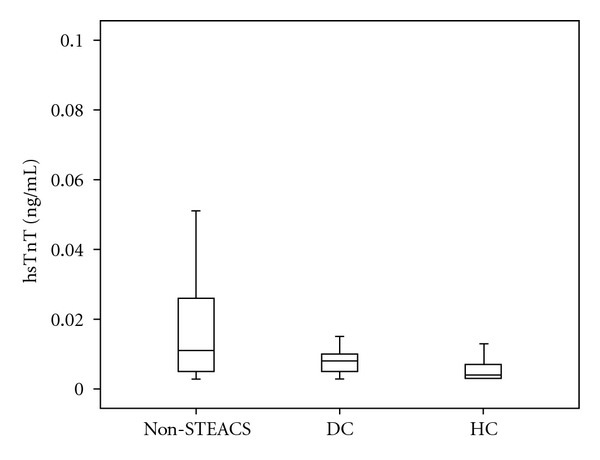
hsTnT levels in non-STEACS, stable coronary disease (disease controls) and healthy controls [values are median (IQR), and non-STEACS: non-ST elevation acute coronary síndrome, DC: disease controls, HC: healthy controls]. *P* < 0.001. [Values are median, IQR, and error bars].

Patients with non-STEACS presented higher serum hsTnT than those with stable CAD and healthy controls [0.010 (0.005–0.023) versus 0.008 (0.005–0.011); *P* = 0.036 and 0.010 (0.005–0.023) versus 0.004 (0.003–0.007); *P* < 0.001, resp.)] (Table 1, Figure 1). There was a positive correlation between hsTnT levels and TIMI risk score (Spearman, *r* = 0.39  *P* < 0.001). There were no differences in hsTnT levels related to current medication on hospital admission (all *P* values > 0.05, Table 2).

At 12 months of followup, 22 (18.0%) patients presented adverse clinical events: 7 (5.7%) cardiovascular death, 3 (2.5%) nonfatal MI, 9 (7.4%) unstable angina, 2 (1.6%) stroke, and 1 (0.8) heart failure. We found a significant association between presenting adverse events and showing positive hsTnT levels [OR : 2.99, 95% IC (1.18–7.57); *P* = 0.021].

There was a significant association between increased hsTnT levels and presenting at least three cardiovascular risk factors, age > 65 years, ST deviation in the ECG, female sex, and angiographic severe lesions [OR = 4.19 (95% CI: 1.76–9.96), *P* = 0.001; OR = 2.40 (95% CI: 1.21–5.12), *P* = 0.024; OR = 5.60 (95% CI: 2.44–12.86), *P* < 0.001; OR = 0.35 (95% CI: 0.15–0.86, *P* = 0.022; OR = 5.54 (95% CI: 2.07–14.83), *P* = 0.001, resp., Table 3]. In the multivariable analysis ST deviation, presenting at least three cardiovascular risk factors, ST deviation, female sex, and severe angiographic lesions remained as independent associated variables with increased hsTnT levels [OR = 3.79 (95% CI: 1.02–14.16), *P* = 0.047; OR = 6.27 (95% CI: 1.98–19.89), *P* = 0.002; OR = 0.17 (95% CI: 0.004–0.68), *P* = 0.013; OR = 6.41 (95% CI: 1.41–29.24), *P* = 0.016, resp., Table 3).

**Figure 2:**
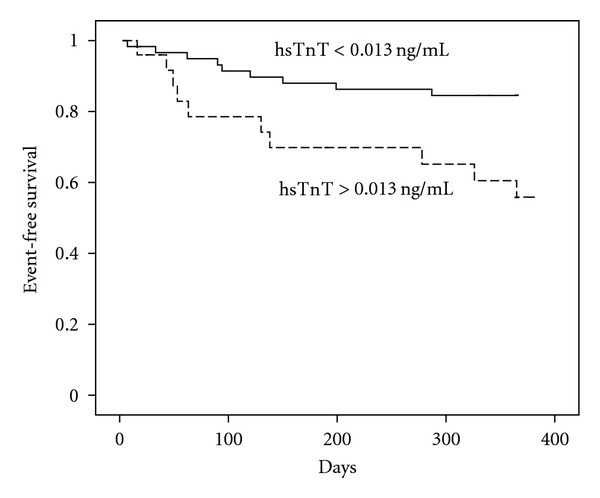
Kaplan-Meier curve showing the effects of hsTnT levels on adverse events following in non-STEACS patients. Patients with raised hs TnT levels, had a significant worse outcome compared with patients with lower hsTnT levels at 12-month followup (cutoff point >0.0013 ng/mL; log-rank test, *P* = 0.06).

According to the established cutoff point for hsTnT in ACS (0.013 ng/mL), we performed univariable Cox regression analysis (Table 4). High serum hsTnT levels [HR 3.29 (95% CI: 1.33–8.11), *P* = 0.010], age >65 years [HR 3.15 (95% CI: 1.20–8.31), *P* = 0.020], and previous non-STEACS [HR 4.35 (95% CI: 1.68–11.26), *P* = 0.002] were significant predictors of adverse outcomes. Kaplan Meier curves show that those with hsTnT levels above the cutoff point presented a worse prognosis (log-rank test, *P* = 0.006; Figure 2). In a multivariable Cox regression analysis, hsTnT and previous non-STEACS remained as independent predictors of adverse outcomes [HR 2.74 (95% CI: 1.08–6.95), *P* = 0.034; HR 4.02 (95% CI: 1.55–10.43), *P* = 0.004, resp.].

We also explored the prognostic implication of hsTnT in the different scenarios, invasive (64 patients) and conservative approach (58 patients). We found a very relevant prognostic implication for hsTnT levels in patients with the conservative approach [HR 11.45 (95% CI: 2.93–44.72), *P* < 0.001], remaining as independent prognostic factor after multivariate analysis (Table 5), whereas this biomarker did not present any relevant implication in the interventional approach in whom coronary catheterism was performed [HR 0.78 (95% CI: 0.20–3.14), *P* = 0.731]. In the multivariate analysis, only high hsTnT levels and presenting previous non-STEACS remained as independent predictors of adverse outcomes at 12-month followup [HR 6.20 (95% CI: 1.31–29.27), *P* = 0.021; HR 12.00 (95% CI: 1.43–100.52), *P* = 0.022, resp.].

### 3.2. Copeptin

Copeptin levels were significantly higher in non-STEACS patients than healthy controls (*P* = 0.021), without differences with disease controls (*P* = 0.186). This biomarker did not show any correlation to classical TnT values obtained at admission (Spearman, *r* = 0.006, *P* = 0.951). Copeptin levels also were not found to be correlated to TIMI risk score (Spearman, *r* = −0.102, *P* = 0.318). We did not find significant association between increased Copeptin levels (above cutoff point of 14 pmol/L) and any of the studied clinical variables, and Cox analyses did not reveal Copeptin as a significant predictor in non-STEACS patients (Table 4) nor in patients undergoing conservative approach (Table 5).

## 4. Discussion

Although many biomarkers have been investigated in recent years to be used for risk stratification in ACS, cardiac Tn still remains as the preferred and really used biomarker for routine differential cardiovascular diagnosis [8, 9, 25]. Cardiac Tn measured by automated standard assays is superior to all other available biomarkers in clinical practice, including myoglobin, the MB fraction of creatine kinase, myeloperoxidase, and heart fatty acid-binding protein, for the diagnosis of acute myocardial infarction [26–28]. However, a considerable number of patients cannot still be identified as being at high risk by available routine biomarkers. New high-sensitive cardiac Tn assays have improved precision at the lower limit of detection and have been shown to be very useful in the early diagnosis of acute myocardial infarction [14], but there is a strong debate about the clinical use and limitations of these new Tn high-sensitive detection methods [22, 29, 30]. A recent study by Bonaca et al. showed that low-level increases in Tn I using a sensitive assay identify patients at higher risk of death or MI among patients with non-STEACS, as a probe of the incremental value of newer, more sensitive assays in identifying high-risk patients with ACS [31]. Our hypothesis is that serum hsTnT levels could play an important role for risk stratification, therapeutic decision-making, and prognosis in non-STEACS patients.

The present study shows that patients with non-STEACS and negative conventional TnT (>0.035 ng/mL) present a rate of 16% of adverse events, with a remarkable 4% rate of death. This highlights that these apparently non- high-risk patients actually require a better risk stratification strategy. Moreover, more than 60% of our included patients were diagnosed with unstable angina or non-Q acute myocardial infarction, pointing out the moderate risk character of our selected population. These findings are in concordance with those previously described in former studies including similar patients profiles [32, 33]. In this sense, we show that our non-STEACS patients present significantly higher levels of hsTnT than controls and chronic stable patients. A recent and complete study by Reichlin et al. [14] reveals that hs-Tn assays can substantially improve early diagnostic and allow treatment options for patients presenting with acute myocardial infarction. Now, we add important findings in the utility of hsTnT, as we propose that hsTnT measurement allows the identification and stratification of patients presenting with non-STEACS and provides relevant information about the prognosis at 12-month followup. Hence, we found that patients with raised serum hsTnT levels (above 0.013 ng/mL) presented more adverse events than those with lower hsTnT levels. Interestingly, raised hsTnT levels maintained its independent prognostic value, together with the ST deviation and previous aspirin treatment, even after adjusting for other consistent variables. In addition, we also show, for the first time, that raised hsTnT levels are independently associated with cardiovascular risk factors and ST deviation in non-STEACS patients, after adjusting for other confounding variables which is of high relevance since hsTnT could identify patients at higher risk of presenting cardiovascular events.

Nowadays the therapeutic decision in non-STEACS is still basically under the criterium of the cardiologist, although guidelines and recommendations appear in continuous revision of the clinical management of these patients [10, 11]. In the same way, we propose that serum hsTnT levels could help in this treatment decision, since in patients who did not underwent catheterization, those showing high levels of serum hsTnT (cut-off point 0.0225 ng/mL) presented a worse outcome. Furthermore, the established cutoff point 0.0130 ng/mL did not predict the outcome for these patients. This emphasizes the utility of this biomarker, especially in patients apparently not elective for cardiac catheterization under classical stratification, in which raised hsTnT levels could be indicative for interventionism recommendation.

In order to evaluate the use of biomarkers of different pathophysiologic profiles, we also tested a biomarker of acute endogenous stress, copeptin. Copeptin was found to present predictive value in coronary artery disease [19], and it has been proposed to be of great value in the rapid rule out of acute MI used in combination with TnT increased levels [15, 19]. We also study the association between the classical clinical and electrocardiographic parameters in order to achieve a better stratification and managements as well as the prognosis of patients with non-STEACS. We detected significant higher serum copeptin concentration in non-STEACS' patients in comparison with healthy controls. However, this difference was not significant when comparing with chronic stable patients. In addition, we were not able to find any significant association with clinical parameters or prognostic implications for serum copeptin. One possible reason for the lack of relevant results with copeptin is its rapid release after symptom onset and the normalization of its levels to below the 99th percentile in 10 hours as prevously reported [16, 20]. Since we collected blood samples within the first 48 h from the pain onset, it is possible that in most of patients, copeptin detected levels did not correspond with the real raised values within the first four-hour window. Additional studies should be done to clarify copeptin relevance in the first 6 h from the pain onset in non-STEACS patients.

Our study has the important limitation of the measurement of single baseline samples in determining prognosis. Although hsTnT levels correlated with TIMI risk score and seems to predict poor outcomes at 12-month followup, we cannot discard changes in hsTnT levels during the time. In addition, the size of our population can also limit the power of our study. Bigger-sized studies are recommended but at this point our data seems to be of great relevance. On the other hand, copeptin levels should be measured within the first four hours form the pain onset in order to reevaluate its clinical relevance in non-STEACS patients.

## 5. Conclusion

In conclusion, serum hsTnT levels are significantly increased in non-STEACS patients and may be involved in the pathogenesis of this condition. High hsTnT levels improve discrimination of patients at risk of adverse events are predictive of adverse events, and should be included in the therapeutic decision-making.

## Figures and Tables

**Table 1:** Baseline characteristics of patients and controls.

	Non-STEACS	Disease controls	Healthy controls
*N*	122	33	43
Age (mean ± SD)	63.2 ± 11.4	63.9 ± 12.7	66.62 ± 7.8
Male sex (%)	83 (68.0)	31 (93.9)	17 (39.5)
Hypertension (%)	92 (75.4)	26 (78.8)	23 (53.5)
Diabetes mellitus (%)	40 (32.8)	14 (42.4)	10 (23.3)
Hypercholesterolemia (%)	67 (54.9)	20 (60.6)	11 (25.6)
Smoking habit (%)	27 (22.1)	—	—
*Medications*		—	—
Aspirin (%)	120 (98.4)	—	—
Clopidogrel (%)	120 (98.4)	—	—
ARBs (%)	34 (27.9)	—	—
Beta-blocker (%)	50 (41.0)	—	—
ACE inhibitors (%)	33 (27)	—	—
CA (%)	25 (20.5)	—	—
Statins (%)	64 (52.0)	—	—
ST deviation (%)	39 (32)	—	—
TIMI risk score (mean ± SD)	2.04 ± 1.31	—	—
Catheterism (%)	64 (52.5)		
Stent carrier (%)	54 (43.9)		
hsTnT (ng/mL)	0.010 (0.005–0.023)	0.008 (0.005–0.011)	0.004 (0.003–0.007)
Copeptin >14 pmol/mL (%)	10 (8.2)	4 (12.1)	1 (2.3)
Copeptin (pmol/L)	8.42 (5.60–13.35)	11.67 (7.08–14.56)	6.51 (4.80–9.08)

High-sensitivity troponin T (hsTnT) and copeptin levels data shown as median (IQR). NSTEACS: non ST-elevation acute coronary syndrome; ARBs: angiotensin receptor blockers; ACE: angiotensin-converting enzyme; CA: calcium antagonists.

**Table 2:** hsTnT levels in relation to different modalities of drug therapy.

Therapy	hsTnT levels (ng/mL)	*P* value
Aspirin	Yes: 0.014 (0.007–0.027)	0.583
	No: 0.008 (0.008–0.008)	
Clopidogrel	Yes: 0.013 (0.001–0.026)	0.383
	No: 0.029 (0.029–0.029)	
ARBs	Yes: 0.009 (0.004–0.016)	0.557
	No: 0.010 (0.005–0.026)	
Beta-blocker	Yes: 0.011 (0.005–0.026)	0.478
	No: 0.009 (0.004–0.021)	
ACE inhibitors	Yes: 0.010 (0.005–0.023)	0.771
	No: 0.009 (0.005–0.024)	
CA	Yes: 0.012 (0.007–0.023)	0.370
	No: 0.009 (0.004–0.021)	
Statins	Yes: 0.013 (0.005–0.029)	0.087
	No: 0.009 (0.004–0.029)	

Values are median (IQR). ARBs: angiotensin receptor blockers; ACE: angiotensin-converting enzyme; CA: calcium antagonists.

**Table 3:** Association of elevated hsTnT levels with clinical features. Logistic regression analyses. Cutoff point for hsTnT = 0.013 ng/mL.

	Univariate		Multivariate	
Condition	OR (95% CI)	*P*	OR (95% CI)	*P*
age > 65 years	2.40 (1.21–5.12)	**0.024**	2.42 (0.78–7.53)	0.127
Previous non-STEACS	1.84 (0.69–4.86)	0.221		
Female sex	0.35 (0.15–0.86)	0.022	0.17 (0.04–0.68)	**0.013**
≥3 CVRF	4.19 (1.76–9.96)	**0.001**	3.79 (1.02–14.16)	**0.047**
Previous ASA taking	1.92 (0.89–4.15)	0.097	0.92 (0.26–3.29)	0.893
Severe symptoms	0.64 (0.31–1.55)	0.396		
ST deviation	5.60 (2.44–12.86)	**<0.001**	6.27 (1.98–19.89)	**0.002**
PCI at admission	1.92 (0.90–4.10)	0.094	2.65 (0.75–9.37)	0.131
Angiographic lesions >50%	5.54 (2.07–14.83)	**0.001**	6.41 (1.41–29.24)	**0.016**

Previous non-STEACS: Previous non-ST elevation acute coronary syndrome. ≥3 CVRF: at least three cardiovascular risk factors. ASA: acetylsalicylic acid. OR: odds ratio. CI: confidence interval. PCI: percutaneous coronary intervention.

**Table 4:** Cox regression analysis at 12-month followup.

	Univariate		Multivariate*	
Condition	HR (95% CI)	*P*	HR (95% CI)	*P*
**hsTnT > 0.013 ng/mL**	3.29 (1.33–8.11)	**0.010**	2.74 (1.08–6.95)	**0.034**
Copeptin > 14 pmol/L	0.04 (0.00–14.78)	0.285		
age > 65 years	3.15 (1.20–8.31)	**0.020**	2.52 (0.88–7.21)	0.111
Severe symptoms	1.65 (0.65–4.17)	0.294		
≥3 CVRF	2.00 (0.75–5.32)	0.167		
Previous non-STEACS	4.35 (1.68–11.26)	**0.002**	4.02 (1.55–10.43)	**0.004**
ST deviation	1.14 (0.32–3.95)	0.841		

^∗^Multivariate analysis by conditional method. ≥3 CVRF: at least three cardiovascular risk factors. Previous non-STEACS: previous non-ST elevation acute coronary syndrome. HR: hazard ratio. CI: confidence interval.

**Table 5:** Cox regression analysis at 12-month followup for patients without invasive catheterization (*n* = 58).

	Univariate		Multivariate	
Condition	HR (95% CI)	*P*	HR (95% CI)	*P*
**hsTnT > 0.013 ng/mL**	11.45 (2.93–44.72)	**<0.001**	6.20 (1.31–29.27)	**0.021**
age > 65 years	1.21 (0.35–4.20)	0.760		
Copeptin > 14 pmol/L	0.039 (0.00–95.09)	0.416		
Severe symptoms	1.18 (0.27–4.74)	0.811		
≥3 CVRF	3.67 (0.98–13.68)	0.053	1.39 (0.31–6.20)	0.667
Previous non-STEACS	17.53 (2.19–140.57)	**0.007**	12.00 (1.43–100.52)	**0.022**
ST deviation	2.78 (0.58–13.44)	0.202		

≥3 CVRF: at least three cardiovascular risk factors. Previous non-STEACS: previous non-ST elevation acute coronary syndrome. HR: hazard ratio. CI: confidence interval.

## Abbreviations

hsTnT: High-sensitivity troponin T
ACS: Acute coronary syndrome
non-STEACS: Non ST-elevation acute coronary syndrome
NT-pro BNP: Amino-terminal pro-B-type natriuretic peptide
CI: Confidence interval
IQR: Interquartile range.
